# Supplementary figures and images for: Somatic Copy Number Alterations in Colorectal Cancer Lead to a Differentially Expressed ceRNA Network (ceRNet)
Source: Curr Issues Mol Biol. 2023 Nov 28;45(12):9549–65. doi: 10.3390/cimb45120597 (PMC10742218; doi:10.3390/cimb45120597)

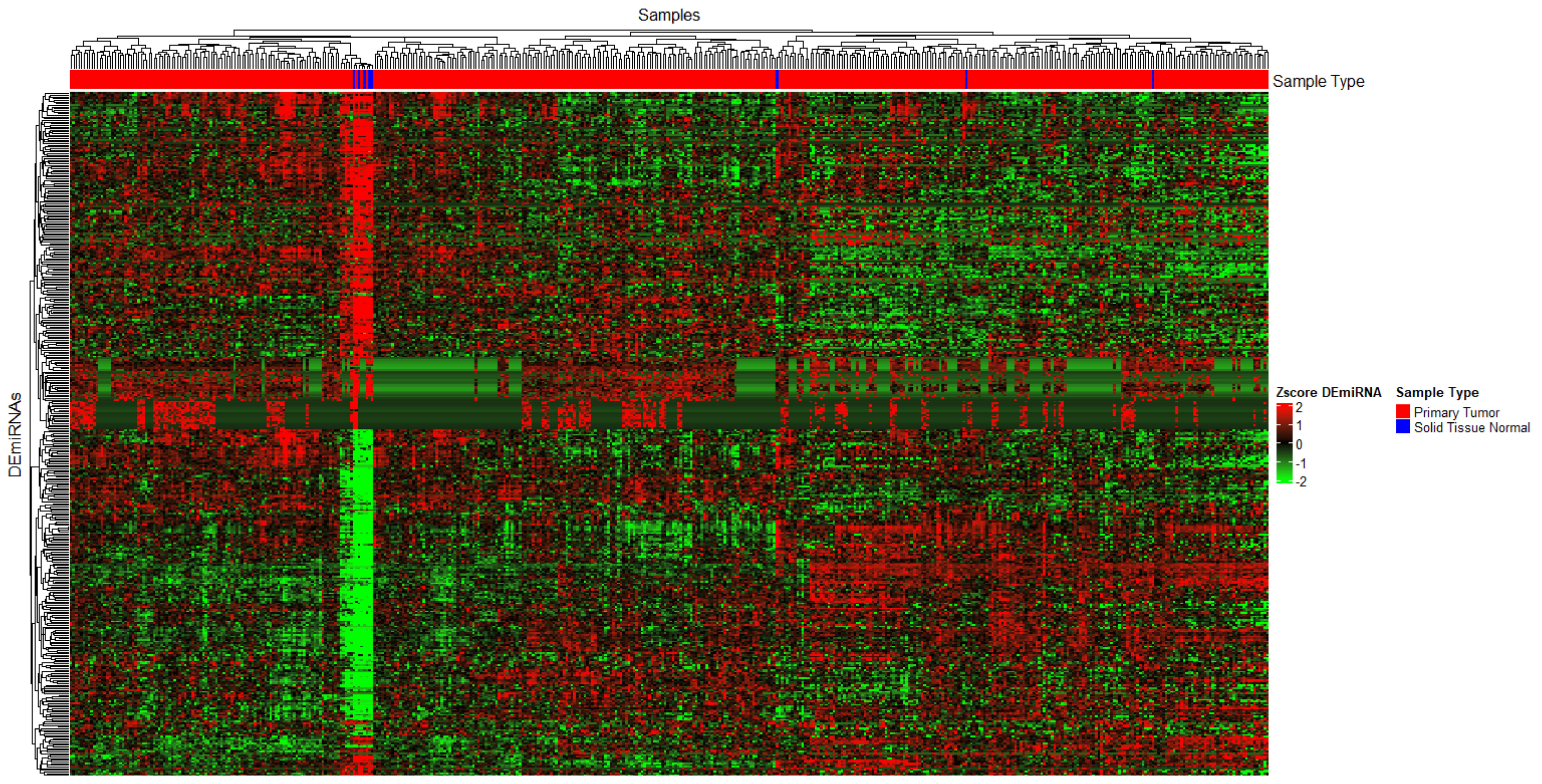

Supplement: Supplementary file 1 [file cimb-45-00597-s001.zip › FigureS1.tif]

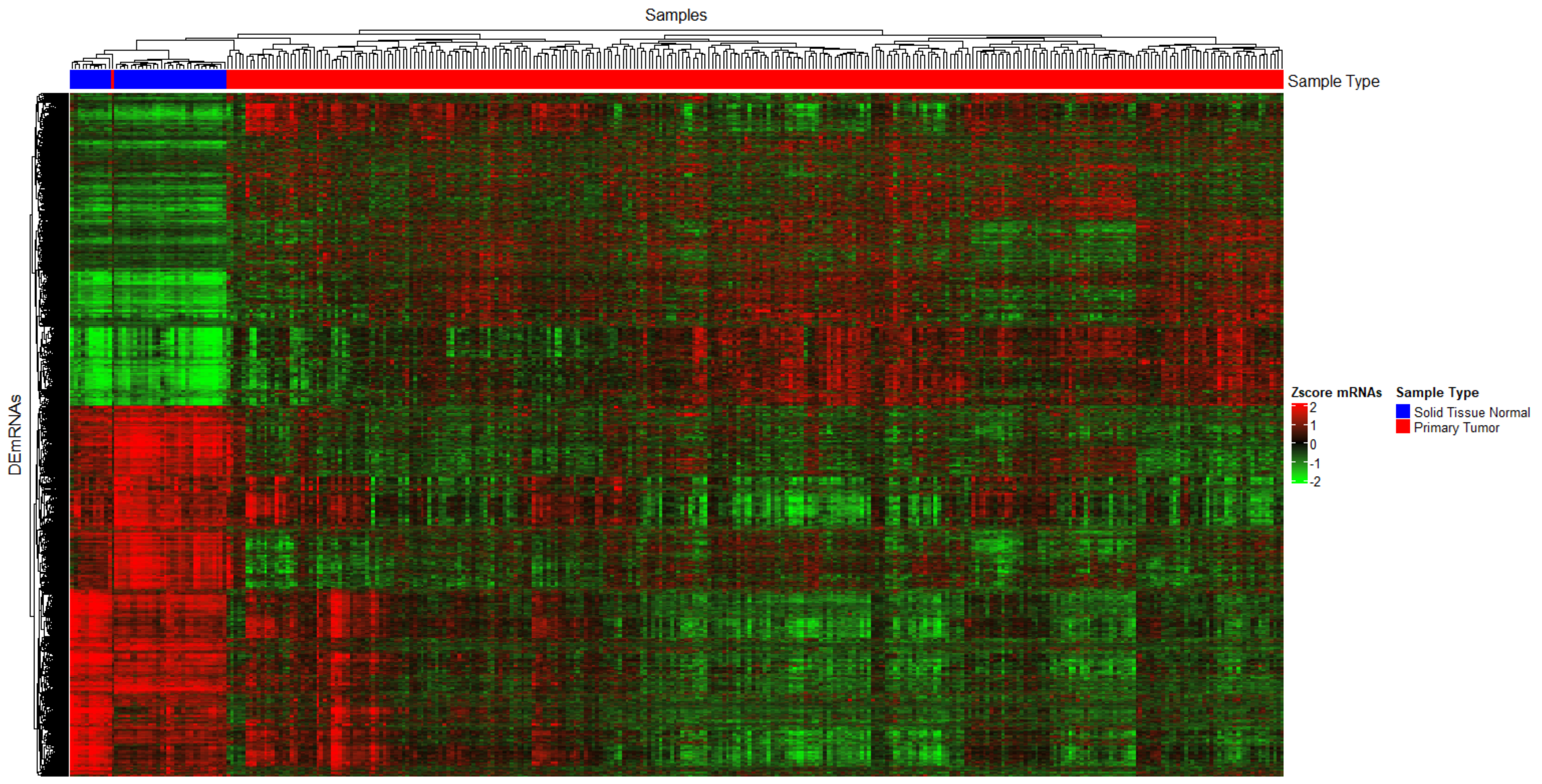

Supplement: Supplementary file 1 [file cimb-45-00597-s001.zip › FigureS2.tif]

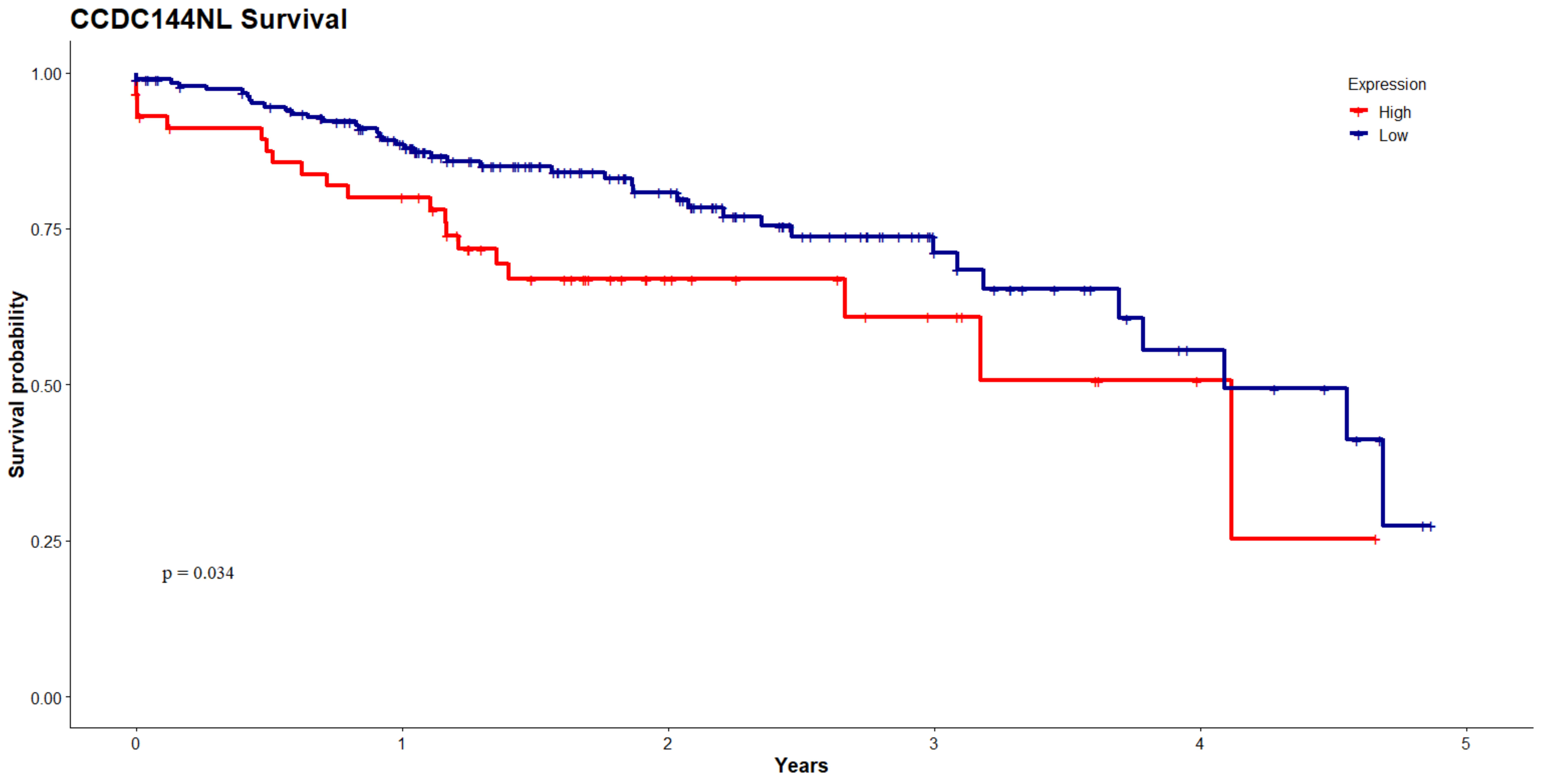

Supplement: Supplementary file 1 [file cimb-45-00597-s001.zip › FigureS3.jpg]

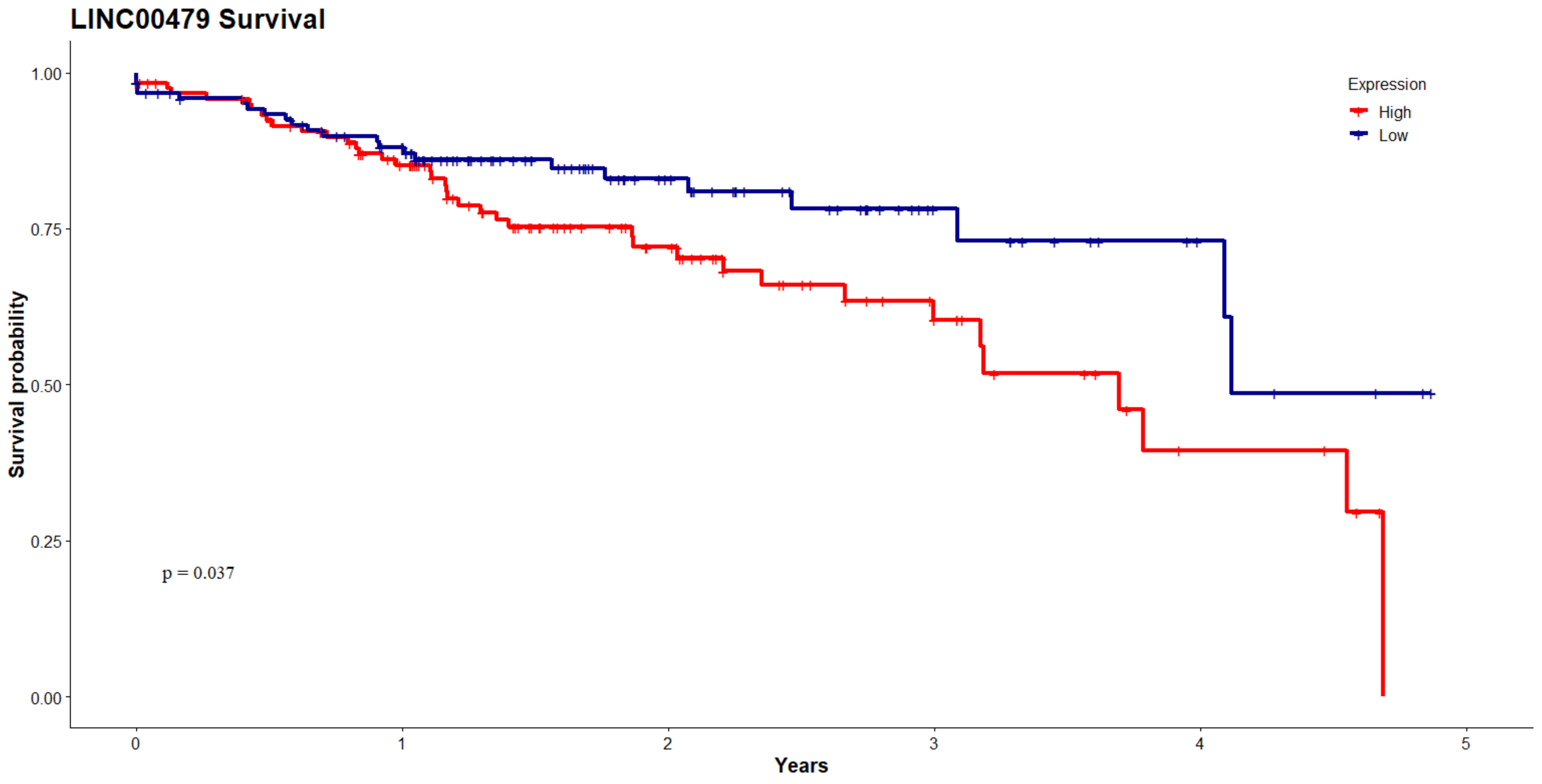

Supplement: Supplementary file 1 [file cimb-45-00597-s001.zip › FigureS4.jpg]

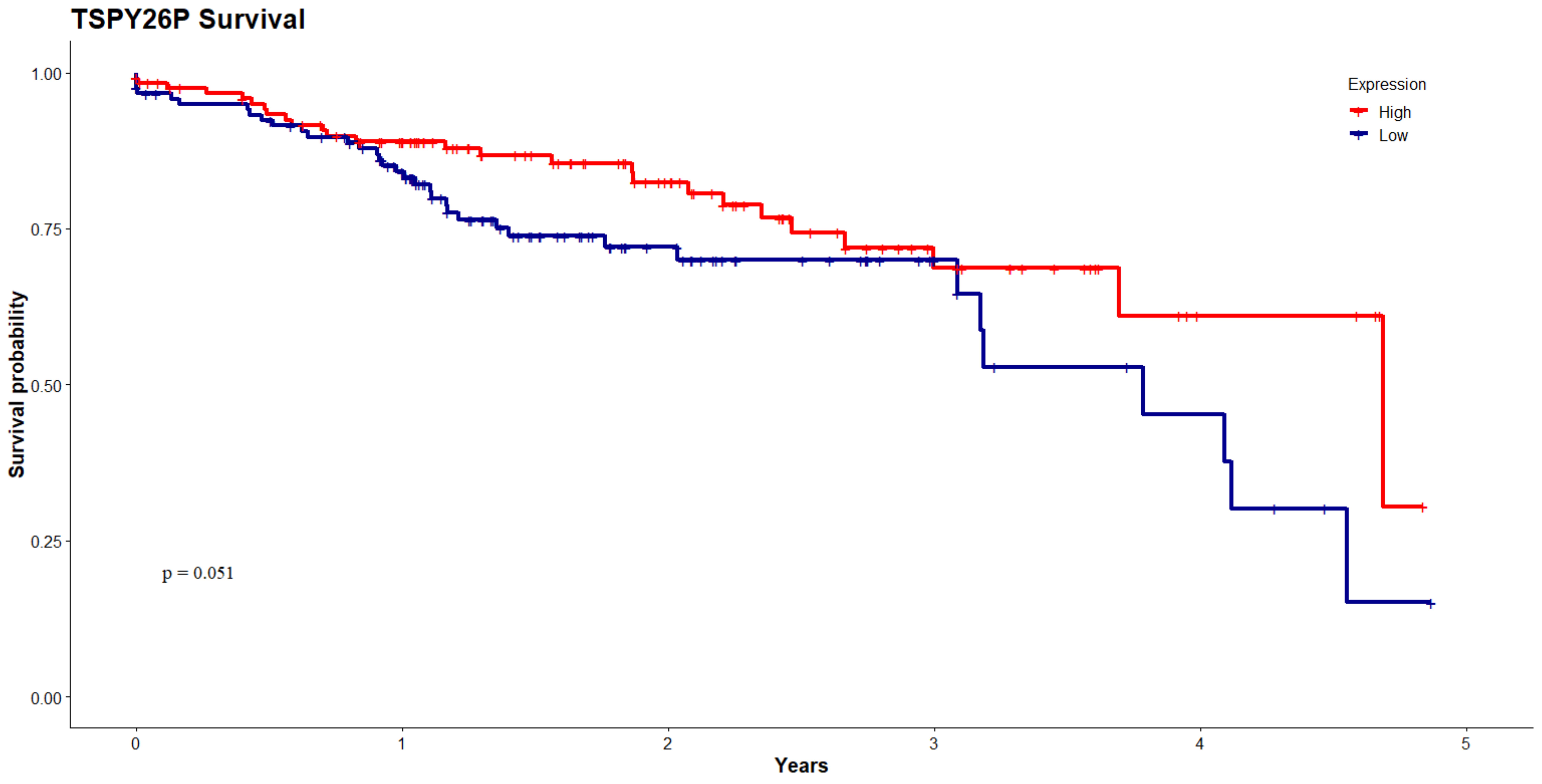

Supplement: Supplementary file 1 [file cimb-45-00597-s001.zip › FigureS5.jpg]

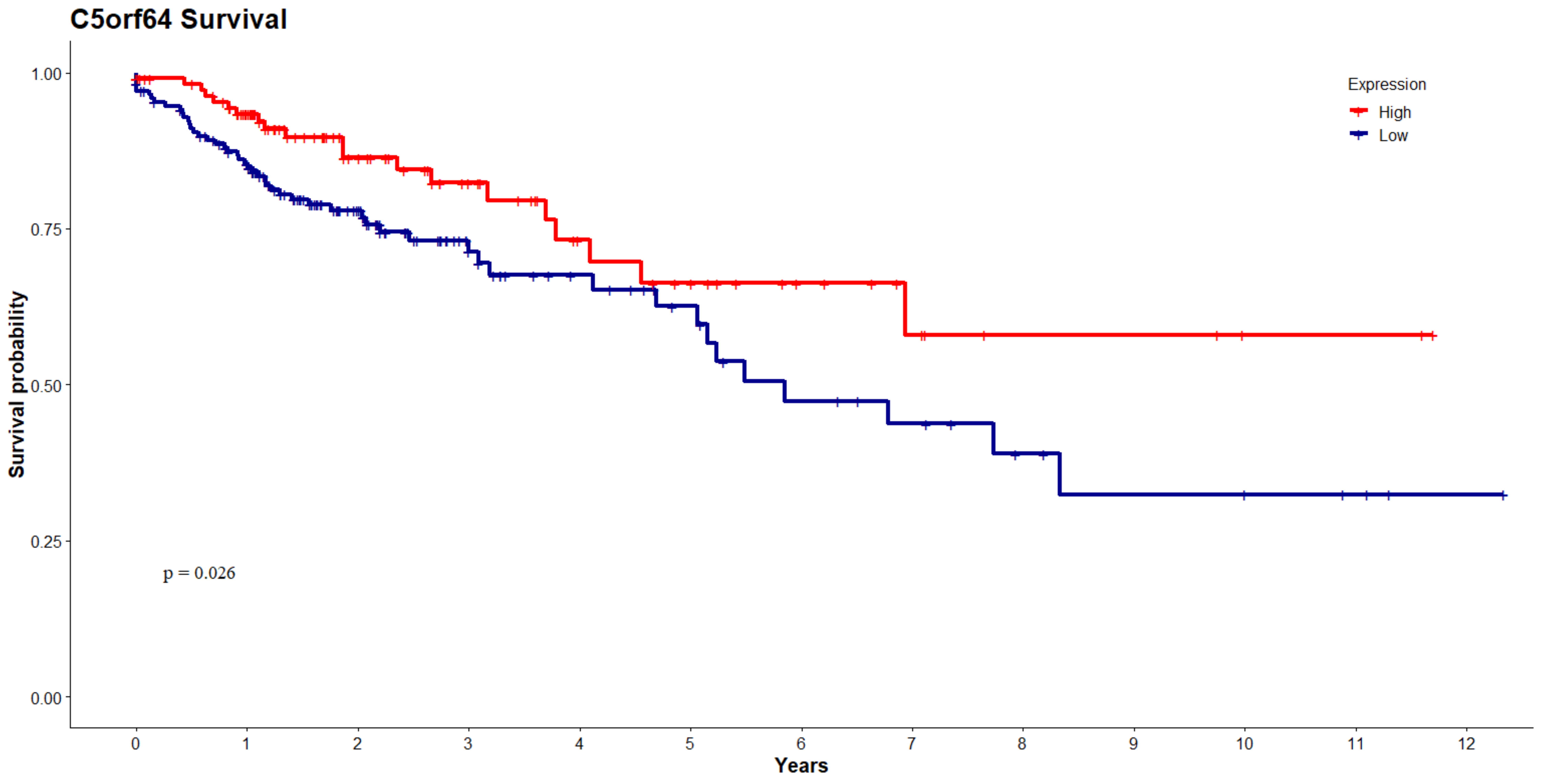

Supplement: Supplementary file 1 [file cimb-45-00597-s001.zip › FigureS6.jpg]
